# Age-related changes in visual performance: A defocus curve study in healthy phakic eyes

**DOI:** 10.1371/journal.pone.0343521

**Published:** 2026-02-20

**Authors:** Inas Baoud Ould Haddi, Águeda Sierra, Nuria Garzón, César Albarrán-Diego, Javier Vargas, María García-Montero

**Affiliations:** 1 Dpto.de Optometría y Visión, Universidad Complutense de Madrid, Madrid, Spain; 2 Departamento de Óptica, Facultad de Ciencias Físicas Universidad Complutense de Madrid, Madrid, Spain; 3 Dpto. de Óptica y Optometría y Ciencias de la Visión, Universitat de València, Burjassot, Spain; Saarland University, GERMANY

## Abstract

This study aimed to characterize the impact of age on visual performance by analyzing monocular defocus curves across a wide age range in healthy phakic eyes. This observational cross-sectional study included 105 subjects, evenly distributed into seven age groups (20–65 years). Monocular visual acuity (VA) was measured at defocus levels from −3.00 D to +1.00 D in 0.50 D steps. Defocus curves were analyzed using four key metrics: baseline VA at zero defocus, curve slope, area under the curve (AUC), and the negative defocus cut-off point at 0.2 logMAR VA. A linear mixed-effects model assessed group differences in defocus curve shape, while non-parametric and parametric tests evaluated differences in AUC and cut-off points. We found that defocus curves remained stable up to 44 years of age, with a significant decline in baseline VA, curve steepness, and overall visual performance beginning at 45 years. AUC values significantly decreased with age, with a clear demarcation between younger (G1–G3; 20–44 years) and older groups (G4–G7; 45–65 years). The 0.2 logMAR cut-off point could not be reached in most younger subjects, indicating preserved accommodative capacity. In contrast, older subjects showed a progressive reduction in depth of focus, with statistically significant differences particularly between groups G4 (45–50 years) and G7 (61–65 years). Overall, these results indicate that defocus curve analysis reveals a functional inflection point in visual performance beginning at 45 years of age. This transition reflects early presbyopic changes, marked by reduced tolerance to defocus and diminished depth of focus. These findings support the use of defocus curves as a valuable tool to assess age-related visual changes beyond conventional distance and near VA tests.

## Introduction

The defocus curve (DC) has proven to be an effective method for assessing visual performance at different distances and, consequently, for evaluating the depth of focus at various ages [1,2]. A common approach to evaluating depth of focus involves measuring the patient’s visual acuity (VA) while different accommodative demands are induced by optically modifying the vergence of a fixed optotype through the use of trial lenses, instead of physically relocating the chart. Defocus curves should be measured considering the manifest refraction with an object at infinity and the vergence correction based on the test distance used for measuring the defocus curve [3]. It is important to note that bringing an object closer to the patient induces a change in light vergence similar to that produced by a spherical lens. This principle underlies the use of defocus lenses, where the dioptric power (D) is calculated as the inverse of the viewing distance in meters (D = 1/–d), allowing simulation of objects at various distances.

Traditionally, defocus curves establish normative references that are useful for evaluating the efficacy of visual correction strategies, such as multifocal contact lenses or intraocular lenses, and for guiding clinical decisions aimed at preserving or restoring near and intermediate vision [4,5]. In fact, DC has been established as the gold standard for evaluating visual acuity provided by an multifocal intraocular lens at different distances, using negative lenses to simulate defocus [6].

However, defocus curve analysis has received less attention in younger, phakic or emerging presbyopic population. This approach provides data not only into corrected distance visual acuity (CDVA), but also into blur tolerance and accommodative performance, contributing to the characterization of visual function across the aging process.

For these reasons, defocus curve analysis across a broad age range of healthy phakic subjects is required to characterize not only visual acuity outcomes but also overall visual performance at different levels of optically induced defocus.

The aim of this study was to characterize the impact of age on visual performance by analyzing monocular defocus curves across a wide age range in healthy phakic eyes.

## Materials and methods

This study was designed as an observational, cross-sectional, and descriptive investigation. Participants were categorized into seven age groups: Group 1 (from 20 to 29 years old), Group 2 (from 30 to 40 years old), Group 3 (from 41 to 44 years old), Group 4 (from 45 to 50 years old), Group 5 (from 51 to 55 years old), Group 6 (from 56 to 60 years old), and Group 7 (from 61 to 65 years old).

In the absence of published data on defocus curves in phakic patients across age groups, we conducted an a priori power analysis based on Cohen’s conventions [7], choosing a large effect size (f = 0.40) as the minimum clinically meaningful difference. Using the R package pwr (pwr.anova.test with k = 7, f = 0.40, α = 0.05, power = 0.80) yielded n = 14 per group (rounded up to 15 for practical reasons).

Participants were recruited at the Complutense University of Madrid (Spain) and evaluated between 10th January and 20th November 2024. All procedures adhered to the ethical principles of the Declaration of Helsinki and were approved by the Ethics Committee of the Hospital Clínico San Carlos in Madrid (Code: 23/039-E). Before participation, all subjects provided written informed consent after receiving a detailed explanation of the study’s objectives and procedures.

The inclusion criteria required participants to be between 20 and 65 years old, have a monocular distance corrected visual acuity (DCVA) of at least 0.10 logMAR, a spherical refractive error from −7.00 to +4.00 D, with astigmatism lower than 1.50 D, and no history of ocular pathology, amblyopia, strabismus, or ocular surgery.

All participants underwent a comprehensive optometric examination, including a routine ocular health assessment, prior to inclusion. Subjects with clinically significant cataract, dry eye disease, accommodative dysfunctions, or any ocular pathology affecting visual function were excluded. Only healthy phakic eyes with normal ocular findings were included. For each subject only the eye with the better visual acuity was considered.

All visual acuity measurements were performed under standardized photopic conditions. The ETDRS chart (Precision Vision, USA) was illuminated to a chart luminance of approximately 85 cd/m^2^, in accordance with recommended clinical standards. Ambient room illumination was maintained at approximately 200–250 lux and kept constant throughout all measurements.

The defocus curve (DC) was assessed in the dominant eye at 4 meters, adding a + 0.25 D lens to compensate for the testing distance. Before testing DC, DCVA was determined through subjective refraction using the Maximum Plus Maximum Visual Acuity (MPMVA) method with the Early Treatment Diabetic Retinopathy Study (ETDRS) chart (Precision Vision, USA) at 4 meters (adding +0.25 D). The DC was assessed using lenses ranging from −3.00 D to +1.00 D in 0.50 D increments. At each level of induced defocus, visual acuity was measured using the ETDRS chart (Precision Vision, USA) at a standardized distance of 4 meters and recorded in logMAR notation with the letter sequences presented in a randomized order.

Ocular dominance was assessed for distance vision using a sensory dominance test [8], which a + 1.50 D lens was alternately placed over each eye while the participant fixated on an ETDRS chart (Precision Vision, USA) placed at 4 meters (distance vision). The eye with which the participant experienced greater visual comfort, despite the induced blur, was classified as non-dominant, while the contralateral eye was considered dominant.

### Statistical analysis

In addition to analysing the full monocular defocus curves, two summary metrics were derived from defocus curves:

- Area Under the Defocus Curve (AUC): the area between the defocus-curve and the 0.20 logMAR reference line was integrated over the range −3.00 D to +1.00 D. Higher AUC values indicate that visual acuity stays ≤ 0.20 logMAR across a wider defocus range, reflecting better overall performance.
- Defocus cut-off at 0.20 logMAR: the negative defocus value (in diopters) at which the defocus curve first crosses the 0.20 logMAR criterion was taken as an estimate the depth-of-focus; larger negative values indicate a wider tolerable defocus range.

#### Analysis of defocus curve differences across age groups.

A linear mixed model (LMM) was chosen instead of a classical ANOVA to statistically compare the defocus curves between age groups. The primary reason for this choice is the repeated-measures nature of the data, since each subject’s visual acuity was measured at multiple defocus levels. Traditional ANOVA assumes independent observations, which is violated due to repeated measurements per subject. LMM accounts for within-subject correlations by including random intercepts, providing a more accurate model.

The LMM analysis was conducted to evaluate the effect of defocus, age group, and their interaction on visual acuity (VA). The model treated defocus (continuous) and age group (categorical) and their interaction (defocus × group) as fixed effects, with subject ID included as a random intercept to account for individual variability. Group 1 (G1) served as the reference category.

After establishing a significant group × defocus interaction, pairwise post-hoc comparisons were carried out to localise the differences between age groups, if they were found. Estimated marginal trends (slopes of VA versus defocus) were compared group-by-group with Holm-adjusted p-values to control the family-wise error rate. These pairwise contrasts identified which specific age groups differed significantly in the steepness or position of their defocus curves.

#### Analysis of the area under the defocus curve, setting a baseline reference at 0.2 logMAR VA.

To quantify visual performance across different defocus levels, we computed the AUC as the integral VA values from the highest positive defocus to the highest negative defocus considered in the study (+1.00 to −3.00 D). Given that VA was measured at discrete 0.50 D defocus steps, a numerical integration approach was necessary.

We used the trapezoidal rule to approximate the integral. This method computes the area as the sum of trapezoidal segments between consecutive defocus values, following the equation:


$$AUC=\sum_{i=1}^{n-1}\frac{\left(y_{i}+\;y_{i+1}\right)}{2}\;\times \;(x_{i+1}-x_{i})$$ (1)

where $x_{i}$ and $x_{i+\text{1}}$ are consecutive defocus levels (in D), and $y_{i}$ and $y_{i+\text{1}}$ are the corresponding VA values (in LogMAR).

This approach ensures that each interval between defocus steps contributes proportionally to the total area based on the local VA values.

To ensure a consistent and meaningful AUC calculation, we set a baseline reference (“floor”) at 0.2 logMAR VA, which represents the lower boundary of functional vision in our study. Any portion of the VA curve below 0.2 logMAR was excluded from the AUC calculation, ensuring that the result reflects only the area where VA remains at or better this threshold.

The area under the defocus curve (AUC) was computed at two hierarchical levels for different purposes.

1. Group-level AUC (descriptive): One value per age group (seven values in total) calculated from the mean defocus curve of each group.
2. Individual-level AUC (inferential): One value for every subject’s monocular defocus curve (15 subjects × 7 groups = 105 values), used to test for between-group differences in AUC.

Individual AUC data normality was assessed using the Shapiro–Wilk test. For normally distributed variables, differences among the seven age groups were examined by one-way Welch’s ANOVA; when Welch’s ANOVA yielded a significant result (p < 0.05), post hoc pairwise comparisons were performed with the Games–Howell test with Holm-adjusted p values to determine which specific group pairs differed. For variables that violated the normality assumption, the non-parametric Kruskal–Wallis test was applied; if it was significant (p < 0.05), Dunn’s test with Holm-adjusted p values was used for post hoc pairwise comparisons. A two-tailed p < 0.05 was considered statistically significant.

#### Analysis of the defocus cut-off point at 0.2 LogMAR VA.

To characterise the depth of focus afforded by the eye in each age group, we identified the negative defocus value (in diopters) at which the monocular defocus curve first crossed the clinical threshold of 0.20 logMAR VA (“cut-off defocus”). Highest negative values denote a wider tolerable defocus range.

Because VA was sampled at discrete 0.50 D steps, the exact crossing rarely coincided with a measured point. We therefore estimated the cut-off by linear interpolation between the two adjacent defocus levels whose VA values straddled the 0.20 logMAR reference:


$$Cut-off=x_{i}+\frac{\left(0.20-\;y_{i}\right)}{\left(y_{i+1}-\;y_{i}\right)}\;\times \;(x_{i+1}-x_{i})$$ (2)

where $x_{i}$ and $x_{i+\text{1}}$ are consecutive defocus levels (in D), and $y_{i}$ and $y_{i+\text{1}}$ are the corresponding VA values (in LogMAR).

Cut-off values were computed at two hierarchical levels, mirroring the AUC analysis:

1. Group-level cut-off (descriptive): One value per age group (7 values) obtained from the *mean* defocus curve of each group.
2. Individual cut-off (inferential): One value for every subject’s monocular defocus curve (15 subjects × 7 groups = 105 values), used to test for between-group differences.

The distribution of individual cut-off values was assessed with the Shapiro–Wilk test. If normality held, Welch’s one-way ANOVA compared the seven age groups; when significant (p < 0.05), Games–Howell post-hoc tests with Holm adjustment located specific group differences. If normality was violated, the Kruskal–Wallis test replaced ANOVA; significant results were followed by Dunn’s pairwise tests with Holm correction. All tests were two-tailed with α = 0.05.

## Results

The study included 105 subjects, evenly distributed across seven age groups (15 subjects per group). Table 1 shows descriptive statistics for age group.

**Table 1:** Descriptive statistics for age (years old), spherical equivalent (D) and sex (% females) by group.

Group	Age (mean)	Age (SD)	Age (max)	Age (min)	SE (mean)	SE (SD)	Sex (% Females)
G1_20–29	23.73	1.81	27	22	−1.38	2.04	66.67
G2_30–40	35.13	3.34	40	31	−1.15	1.83	66.67
G3_41–44	43.00	1.22	44	41	−0.36	0.72	53.33
G4_45–50	48.13	1.55	50	45	−1.38	1.66	53.33
G5_51–55	53.07	1.34	55	51	−0.31	1.35	53.33
G6_56–60	57.87	1.46	60	56	0.23	1.50	73.33
G7_61–65	62.67	1.30	65	61	−1.38	2.93	73.33

SD: standard deviation; SE: spherical equivalent.

### Defocus curve differences among age groups

**Fig 1:**
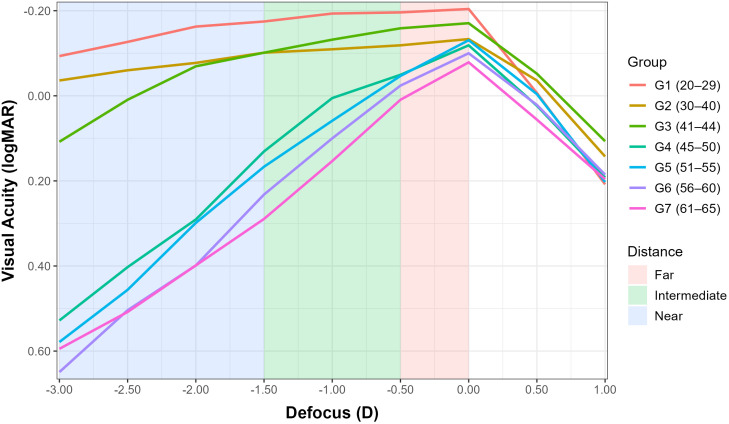
Mean defocus curves for each group.

The mean defocus curves for each group are shown in Fig 1 and the results of linear mixed model analysis are shown in Table 2.

**Table 2:** Linear Mixed Model results of defocus curve for age groups.

Term	Estimate	Std. Error	DF	t value	p value
(Intercept)	−0.057	0.020	169.3	−2.798	0.006
Defocus	0.049	0.010	833	4.861	<0.001
G2 (30–40)	0.020	0.029	169.3	0.692	0.490
G3 (41–44)	−0.009	0.029	169.3	−0.325	0.746
G4 (45–50)	0.097	0.029	169.3	3.374	<0.001
G5 (51–55)	0.100	0.029	169.3	3.501	<0.001
G6 (56–60)	0.123	0.029	169.3	4.299	<0.001
G7 (61–65)	0.154	0.029	169.3	5.351	<0.001
Defocus x G2 (30–40)	−0.027	0.014	833	−1.891	0.059
Defocus x G3 (41–44)	−0.064	0.014	833	−4.495	<0.001
Defocus x G4 (45–50)	−0.165	0.014	833	−11.625	<0.001
Defocus x G5 (51–55)	−0.181	0.014	833	−12.743	<0.001
Defocus x G6 (56–60)	−0.201	0.014	833	−14.158	<0.001
Defocus x G7 (61–65)	−0.188	0.014	833	−13.283	<0.001

DF: degrees of freedom.

A linear mixed-effects model was fitted with logMAR visual acuity as the dependent variable, Defocus (continuous, D) and Age-Group (categorical, 7 levels) as fixed effects, and their interaction (Defocus × Group). A random intercept for subject ID accounted for the correlation of repeated measures.

The Defocus main effect was significant (Estimate = 0.049 logMAR/ D, Std. Error = 0.0100, t = 4.86, p < 0.001), confirming that acuity worsens as defocus increases.

Compared with the reference group (G1 = 20–29), baseline acuity at zero defocus was significantly poorer in groups G4, G5, G6 and G7 (all p ≤ 0.001, see Table 2), while no difference was detected for G2 or G3 (p ≥ 0.490, see Table 2).

Most importantly, the significant Defocus × Group interactions indicate that the slope of the defocus curve differs between age groups, i.e., older groups show a steeper visual-acuity loss with defocus, whereas younger groups (G1 and G2) showed a more gradual VA decline.

Because the interaction was significant, pairwise comparisons of the VA-versus-Defocus slopes were performed (Holm-adjusted). The contrasts (Table 3) show that the slopes for each older group (G3–G7) are significantly steeper than that of G1 (all p < 0.001). No significant difference was found between G1 and G2 (p = 0.354), nor between G2 and G3 (p = 0.075). Within the older age bands, no statistically significant differences were observed among groups G4 to G7 (all p ≥ 0.081).

**Table 3:** Pairwise comparisons of Visual Acuity~Defocus slopes for age group.

Contrast	Estimate	Std. Err	DF	t value	p value
(G1_20–29) – (G2_30–40)	0.027	0.014	833	1.891	0.354
(G1_20–29) – (G3_41–44)	0.064	0.014	833	4.495	<0.001
(G1_20–29) – (G4_45–50)	0.165	0.014	833	11.625	<0.001
(G1_20–29) – (G5_51–55)	0.181	0.014	833	12.743	<0.001
(G1_20–29) – (G6_56–60)	0.201	0.014	833	14.158	<0.001
(G1_20–29) – (G7_61–65)	0.188	0.014	833	13.283	<0.001
(G2_30–40) – (G3_41–44)	0.037	0.014	833	2.603	0.075
(G2_30–40) – (G4_45–50)	0.138	0.014	833	9.734	<0.001
(G2_30–40) – (G5_51–55)	0.154	0.014	833	10.852	<0.001
(G2_30–40) – (G6_56–60)	0.174	0.014	833	12.267	<0.001
(G2_30–40) – (G7_61–65)	0.161	0.014	833	11.392	<0.001
(G3_41–44) – (G4_45–50)	0.101	0.014	833	7.131	<0.001
(G3_41–44) – (G5_51–55)	0.117	0.014	833	8.249	<0.001
(G3_41–44) – (G6_56–60)	0.137	0.014	833	9.663	<0.001
(G3_41–44) – (G7_61–65)	0.125	0.014	833	8.788	<0.001
(G4_45–50) – (G5_51–55)	0.016	0.014	833	1.118	0.791
(G4_45–50) – (G6_56–60)	0.036	0.014	833	2.533	0.081
(G4_45–50) – (G7_61–65)	0.023	0.014	833	1.658	0.489
(G5_51–55) – (G6_56–60)	0.020	0.014	833	1.415	0.630
(G5_51–55) – (G7_61–65)	0.008	0.014	833	0.539	0.791
(G6_56–60) – (G7_61–65)	−0.012	0.014	833	−0.875	0.791

DF: degrees of freedom.

### Area under the defocus curve setting a baseline reference at 0.2 logMAR VA differences among age groups

**Fig 2:**
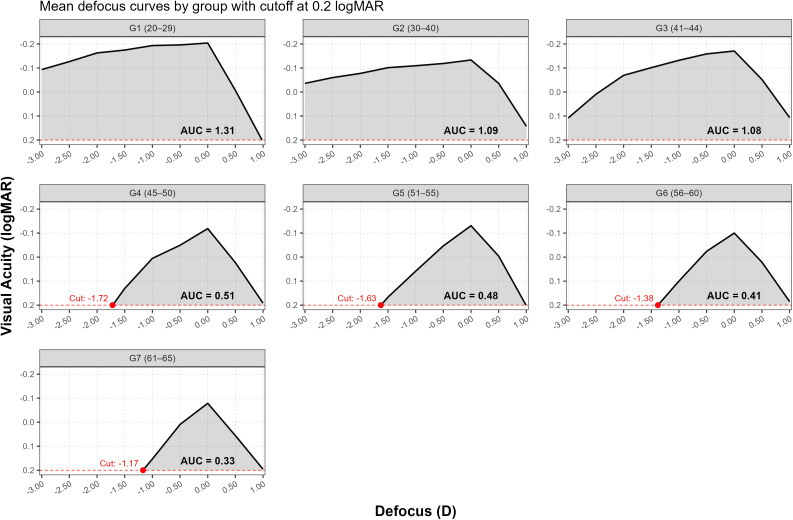
Area under the mean defocus curve for each group (from −3.00 D to +1.00 to) setting baseline reference at 0.2 logMAR VA.

Fig 2 shows the area under the mean defocus curve for each group from +1.00 to −3.00 D and setting baseline reference at 0.2 logMAR VA.

Table 4 shows the mean and standard deviation values for the AUC within each group, for defocus curve measured from +1.00 to −3.00 D, and setting baseline reference at 0.2 logMAR VA.

**Table 4:** Mean ± standard deviation AUC and defocus cut-off with 0.2 LogMAR VA values for each group.

Group	Mean_AUC	SD_AUC	Mean_Cut	SD_Cut	n_Cut
G1_20–29	1.32	0.29	–	–	0
G2_30–40	1.09	0.18	–	–	0
G3_41–44	1.11	0.18	−2.66	0.31	4
G4_45–50	0.53	0.14	−1.78	0.41	15
G5_51–55	0.52	0.17	−1.62	0.50	15
G6_56–60	0.43	0.12	−1.42	0.39	15
G7_61–65	0.37	0.11	−1.26	0.40	15

AUC: area under the curve, SD: standard deviation, Cut: defocus cut-off at 0.2 LogMAR VA level, n_Cut: number of eyes in each group with defocus curves intersecting the 0.2 LogMAR VA level.

**Fig 3:**
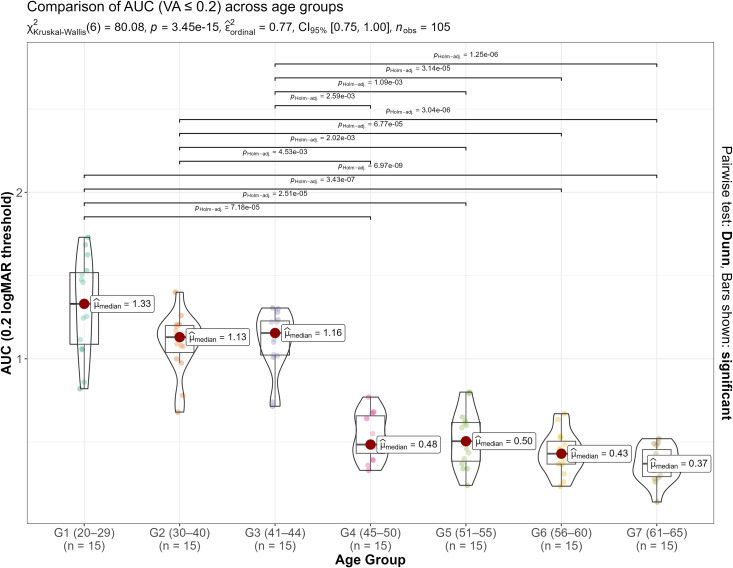
Statistical comparison among age groups for the areas under the defocus curves for each group.

Since AUC data did not pass the normality testing for all groups (Shapiro-Wilk W = 0.868, p = 0.0316 for Group 4), a Kruskal-Wallis test was used instead of a parametric ANOVA to look for differences in AUC among age groups. As can be seen from Fig 3, statistically significant differences were found (p < 0.001).

Post hoc pairwise comparisons (Dun test with Holm adjustment) revealed a clear division of the age groups into two statistically different clusters. Younger groups (G1, G2, and G3) did not differ significantly from each other, yet they showed statistically significant differences compared to each of the older groups (G4 to G7). Similarly, the older groups did not differ significantly among themselves.

### Defocus cut-off point at 0.2 LogMAR VA differences among age groups

Table 4 shows the mean and standard deviation values for the defocus at which defocus curve measured from −3.00 D to +1.00 D cuts with 0.20 LogMAR VA for each group, with “n_Cut” indicating the number of eyes in each group in which the defocus curve cuts the 0.2 LogMAR VA level.

G1 and G2 groups had no eye with a defocus curve cutting 0.2 LogMAR VA level. G3 had only 4 eyes. For this reason, those younger groups with no (enough) defocus cut-off point at 0.2 LogMAR VA values were excluded from the statistical comparison.

**Fig 4:**
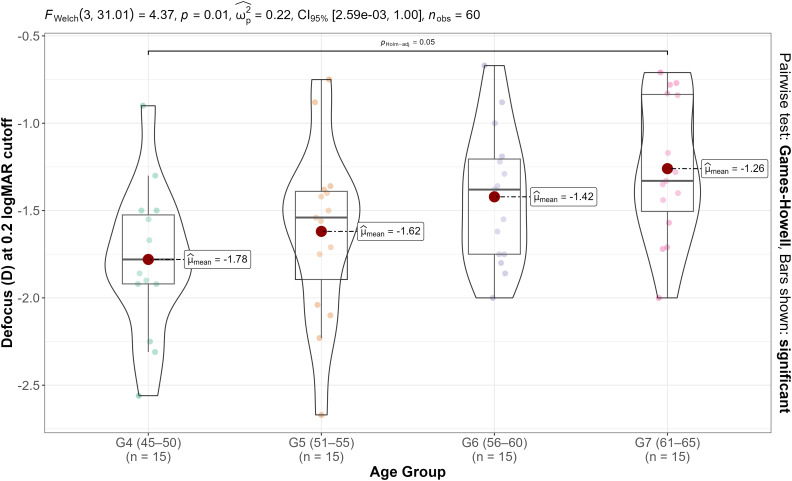
Statistical comparison among age groups for the defocus cut-off values of the defocus curves with the 0.2 LogMAR VA for each group.

Defocus cut-off data pass the normality testing for all groups (G4-G7) (Saphiro-Wilk, p > 0.12 for all groups), so a parametric test could be employed. The One-way Welch’s ANOVA revealed significant differences in the defocus cut-off point at 0.2 LogMAR VA among groups, as can be seen from Fig 4. In addition, post hoc pairwise comparisons (Games-Howell test with Holm adjustment) indicated significant differences between G4 (45–50) and G7 (61–65), while no statistically significant differences were found among other groups, as can be seen from Fig 4.

### Influence of baseline refractive error on defocus curve outcomes

To assess whether baseline refractive error influenced defocus curve outcomes, an additional linear mixed-effects model was fitted including spherical equivalent as covariate. Model comparison using a likelihood ratio test revealed no improvement in model fit with the addition of SE (χ^2^ = 0.014, p = 0.905), and both AIC and BIC values slightly worsened, as can be seen in the following table. These results indicate that SE did not contribute additional explanatory power beyond the effects already captured by defocus and age group. (Table 5).

**Table 5:** Linear mixed-effects model including baseline refractive error as covariates (spherical equivalent and astigmatism).

Model	AIC	BIC	logLik	−2*LOG(L)	Chisq	DF	p-value
baseline_model	−817,08	−739,46	424,54	−849,08	0,014	1	0,905
extended_model	−815,09	−732,62	424,55	−849,09			

AIC: Akaike Information Criterion; BIC: Bayesian Information Criterion; logLik: log-likelihood; Chisq: chi-square statistic from the likelihood ratio test; DF: degrees of freedom.

## Discussion

Despite growing interest in age-related visual changes, defocus curves have rarely been applied across a broad age range of healthy phakic eyes. Yet, their application provides a functional assessment of visual performance that goes beyond what isolated far and near visual acuity measures can reveal. To our knowledge, this is the first study to present monocular defocus curves across such a wide age span in phakic subjects, offering a detailed functional profile of how visual performance changes throughout adulthood.

There are multiple approaches to analyzing and interpreting defocus curve results. In line with the recommendations of Kohnen et al. [9] the present study have employed a range of complementary metrics and statistical methods to comprehensively characterize the defocus curve across a broader age range in phakic eyes, moving beyond the conventional point-by-point comparison. Concretely, the analysis of defocus curves included four metrics: baseline visual acuity at zero defocus, slope of the curve, AUC, and the defocus cut-off point at 0.2 logMAR VA.

The analysis of the defocus curve based on baseline visual acuity at zero defocus provides an estimate of corrected distance visual acuity (CDVA) at optical infinity. It remained stable until approximately 44 years of age, after which a significant decline is observed.

This decline in CDVA has also been reported by other authors who assessed visual acuity in large cohorts of healthy, phakic subjects across a broad age range— though not specifically using defocus curve analysis, and with varying age-related break-points.

The current results are consistent with findings by Sjöstrand, Laatikainen et al. (2011) [10] that found that visual acuity remained stable up to around 43 years in healthy individuals aged 18–88 from Scandinavian populations. Other authors have identified the break-point at around 50 years of age after analyzing a population aged 18–80 years [11].

In contrast, Radner and Benesch (2019) [12] observed a significant decline between ages 55 and 59 in subjects aged 25–74, with CDVA remaining until age 54.

From the analysis of defocus curves, the results of Shafer, Puls-Boever et al. (2023) [1] in healthy, phakic subjects aged 37–48 years showed no change at the 0.00 D defocus level. However, these results were obtained under binocular rather than monocular conditions.

The variability in reported CDVA break-points across studies may reflect differences in visual acuity measurement methods, health status control within study populations, and individual variability in neural processing.

The effect of ageing on visual acuity is well known and is attributed to neuronal loss, increased neural variability, optical degradation due to loss of lens transparency and higher-order aberrations, as well as reduced retinal and cortical efficiency [13,14].

However, CDVA analysis alone does not provide a comprehensive view of the progressive changes in visual acuity with increasing defocus, which offers a more complete perspective on overall visual performance.

So, the analysis of the slope of the defocus curve offered a broader view beyond the single point of visual acuity at distance vision. Younger groups (G1 and G2: 20–40 years) maintained a shallow, non-significant slope, reflecting minimal loss of VA with defocus. A steeper decline emerged from 41–44 years (G3), with pairwise comparisons confirming that the most significant slope change occurred starting at 45 years (G4), exhibiting a parallelism with the break-point for the baseline VA at zero defocus metric.

According to our results, Shafer, Puls-Boever et al. (2023) [1] also reported changes in defocus curves at age 46. However, their analysis did not address the overall slope of the curve, but rather focused on a point-by-point analysis, observing changes in intermediate and near VA corresponding to defocus levels of –1.00 D, –1.50 D, and –2.00 D.

To establish a functional reference standard for visual performance, we analyzed the AUC, using a baseline reference of 0.2 logMAR VA, which represents the lower threshold of functional vision selected for our study. Although cut-off limits are often arbitrary and vary between studies, the 0.2 logMAR is a commonly recommended standard [9,15].

A key aspect of the current AUC analysis is the choice of integration method. While spline interpolation could be used to estimate a continuous function prior to integration, we opted for the trapezoidal rule due to the limited number of defocus steps available.

The AUC significantly worsens starting at age 45 (G4). In contrast, it remains stable between the ages of 20 and 44 (G1, G2, and G3). These results suggest that the AUC parameter can effectively differentiate between younger (20–44 years; G1-G3) and older age groups (45 years and above; G4-G7), but it does not effectively discriminate within these two broader clusters. To our knowledge, AUC values has not been assessed previously in healthy phakic subjects across a broad age range.

In order to quantify the dioptric range over which subjects maintain a clinically level of visual acuity, the present study focused on the analysis of the defocus cut-off point at 0.2 logMAR VA. This value reflects the range-of-focus—also referred to as depth-of-focus [9,16]—which defines the dioptric range over which visual acuity remains equal or better than 0.2 LogMAR.

The evaluated defocus range, from –3.00 D to +1.00 D, was not sufficient to determine the cut-off point at 0.2 logMAR in the younger groups (20–44 years; G1 to G3), as visual acuity remained better than 0.2 logMAR throughout the entire range, likely due to the accommodative capacity in these age groups.

In contrast, in the older groups, it was possible to set the defocus cut-off point at 0.2 logMAR (represented by setting baseline reference at 0.2 logMAR VA, as shown in Fig 2), which progressively decreased with age. According to the inverse of the dioptric value, subjects in these age groups were able to maintain a clear range of vision (up to the threshold of 0.2 logMAR visual acuity) from optical infinity to a distance of approximately 0.58 m (1/1.72 D), 0.61 m (1/1.63 D), 0.72 m (1/1.38 D), and 0.85 m (1/1.17 D) for Groups G4, G5, G6, and G7, respectively. This indicates that with increasing age, the near limit of clear vision progressively shifts further away, reflecting a reduction in the depth of focus.

In fact, depth of focus (represented by the mean cut-off values shown in Table 4 and Fig 4) demonstrated a progressive decline with age across these older groups (45–65 years; G4 to G7). Although some intergroup variability was observed, the most marked and statistically significant reduction occurred between G4 (45–50 years) and G7 (61–65 years), underscoring the extent of functional decline in this 20-year period.

Similar findings have been reported by other authors [17] who examined the dioptric range in healthy phakic subjects aged 40–70 years. Their study included 30 participants per decade (40s, 50s, 60s, and 70s), and mean depth of focus values for the 40s, 50s, and 60s were 1.88 ± 0.77 D, 1.45 ± 0.43 D, and 0.98 ± 0.43 D, respectively. These results, consistent with ours, also demonstrate an age-related reduction in depth of focus. However, their reported values were slightly lower, which may be attributed to differences in methodology. Specifically, they calculated the mean diopters of the accommodation region at which subjects achieved a corrected visual acuity of 20/29 (~0.18 logMAR) that was assessed using a vision tester (AS-15, Kowa Co., Ltd.), by placing only negative spherical lenses [17].

The functional shift observed in defocus curve parameters from 45 years of age is consistent with previously reported inflection points in molecular aging. In particular, a study identified key physiological transitions around the mid-forties, reinforcing the idea that this age represents a critical turning point in both visual performance and systemic biological aging [18].

The methodological procedure applied in this study to analyze the metrics of the defocus curve improves the quality of the clinical data. The use of standardized protocols and focused analytical strategies enables consistent inter-study comparisons and facilitates a more accurate assessment of visual function metrics.

Given that fully corrected ametropes may differ from emmetropes in their accommodative behavior, we tested whether baseline spherical equivalent (SE) influenced the defocus response by including it as a covariate in an extended LMM. However, the model comparison showed no improvement in fit, and SE was not a significant predictor. This supports the robustness of our main model specification and suggests that baseline refractive error, when corrected, does not meaningfully impact defocus curve performance in this healthy phakic sample.

Several limitations of this study should be acknowledged. First, defocus curves were assessed monocularly and under standardized photopic conditions, which allowed isolation of ocular-level visual performance but does not capture binocular visual experience. Second, optical factors such as pupil diameter and higher-order aberrations were not directly measured and may have contributed to interindividual variability, although all participants had healthy eyes and were fully corrected for lower-order aberrations. Finally, sex-related differences in visual performance were not specifically examined. While the sample size was adequate to detect age-related effects, further stratification by both age group and sex would have resulted in small subgroup sizes and reduced statistical power. Future studies with larger cohorts will be needed to investigate potential gender-related differences in defocus curve characteristics across the lifespan.

## Conclusions

The analysis of defocus curves across a broad age range, using four metrics (baseline visual acuity at zero defocus, slope of the curve, AUC, and the defocus cut-off point at 0.2 logMAR VA) reveals a functional shift beginning at 45 years of age.

While all metrics remain stable between the ages of 20 and 44, reflecting the effectiveness of accommodation system and the young visual system’s natural tolerance to minor optical blur, from 45 years onward a clear inflection point appears, characterized by a significant decline in visual performance under defocus conditions.

This indicates a reduced capacity to compensate for defocus and aligns with the early stages of accommodative decline. These findings support the notion that age 45 marks a critical threshold in the visual system’s ability to tolerate blur. This transition marks the onset and progression of presbyopia and reflects a decline in both accommodative amplitude and functional depth of focus.
